# Skeletal muscle metabolomic markers underlying the enhanced exercise-induced hypertrophy response to resistance training in older adults

**DOI:** 10.1007/s11357-025-02074-x

**Published:** 2026-01-05

**Authors:** Changhyun Lim, Manoel Lixandrão, Dakshat Trivedi, Yun Xu, Konstantinos Prokopidis, Hamilton Roschel, Stuart M. Phillips, Howbeer Muhamadali, Masoud Isanejad

**Affiliations:** 1 Faculty of Medical Sciences, Population Health Sciences Institute, Newcastle University, Newcastle Upon Tyne, UK; 2 Department of Kinesiology, McMaster University, Hamilton, ON Canada; 3 Applied Physiology and Nutrition Research Group—School of Physical Education and Sport and School of Medicine, University of São Paulo, São Paulo, Brazil; 4 Centre for Metabolomics Research, Department of Biochemistry, Cell and Systems Biology, Institute of Systems, Molecular and Integrative Biology, University of Liverpool, Liverpool, UK; 5 Department of Musculoskeletal Ageing and Science, Institute of Life Course and Medical Sciences, University of Liverpool, Liverpool, UK; 6 Department of Sport and Exercise Science, Institute of Sport, Manchester Metropolitan University, Manchester, UK

**Keywords:** Ageing muscle metabolism, Resistance training, Muscle hypertrophy

## Abstract

Resistance training (RT) is an effective intervention for improving muscle health and metabolism in ageing, but the degree of responsiveness (hypertrophy) to RT varies substantially. We examined muscle metabolomic profiles before and after 10-weeks RT in older adults classified into upper (UPPER) and lower (LOWER) tertiles of hypertrophy to identify key metabolic adaptation differences. Fifty older adults (23 males, 27 females, mean 68.2 years old) completed 10 weeks of RT combined with whey protein supplementation. Quadriceps cross-sectional area (CSA) was assessed via magnetic resonance imaging before and after RT. Participants were grouped into UPPER (n = 25, 10.3 ± 2% CSA increase) or LOWER (n = 25, 3.3 ± 2% CSA increase) based on ranked CSA changes. We profiled skeletal muscle tissues from the UPPER and LOWER groups using a metabolomics platform. Over 2,500 metabolites were mapped to 104 metabolic pathways. In the UPPER group, upregulation of tryptophan-indole metabolites and the kynurenine pathway suggests a potential role of gut function and anti-inflammatory effect on RT-induced hypertrophy. Also, leucine, isoleucine and valine were significantly upregulated in the absence of their catabolites. Enrichment of urea cycle/amino group metabolism alongside mitochondria-matrix metabolites in the UPPER group indicates improved amino acids and energy homeostasis. Our findings highlight distinct RT-induced skeletal muscle metabolic profiles between UPPER and LOWER in older adults, underscoring the value of metabolic data. These metabolic pathways are important for understanding what contributes to the heterogeneity of hypertrophic response to RT in older adults.

**Graphical Abstract:**

**Figure:**
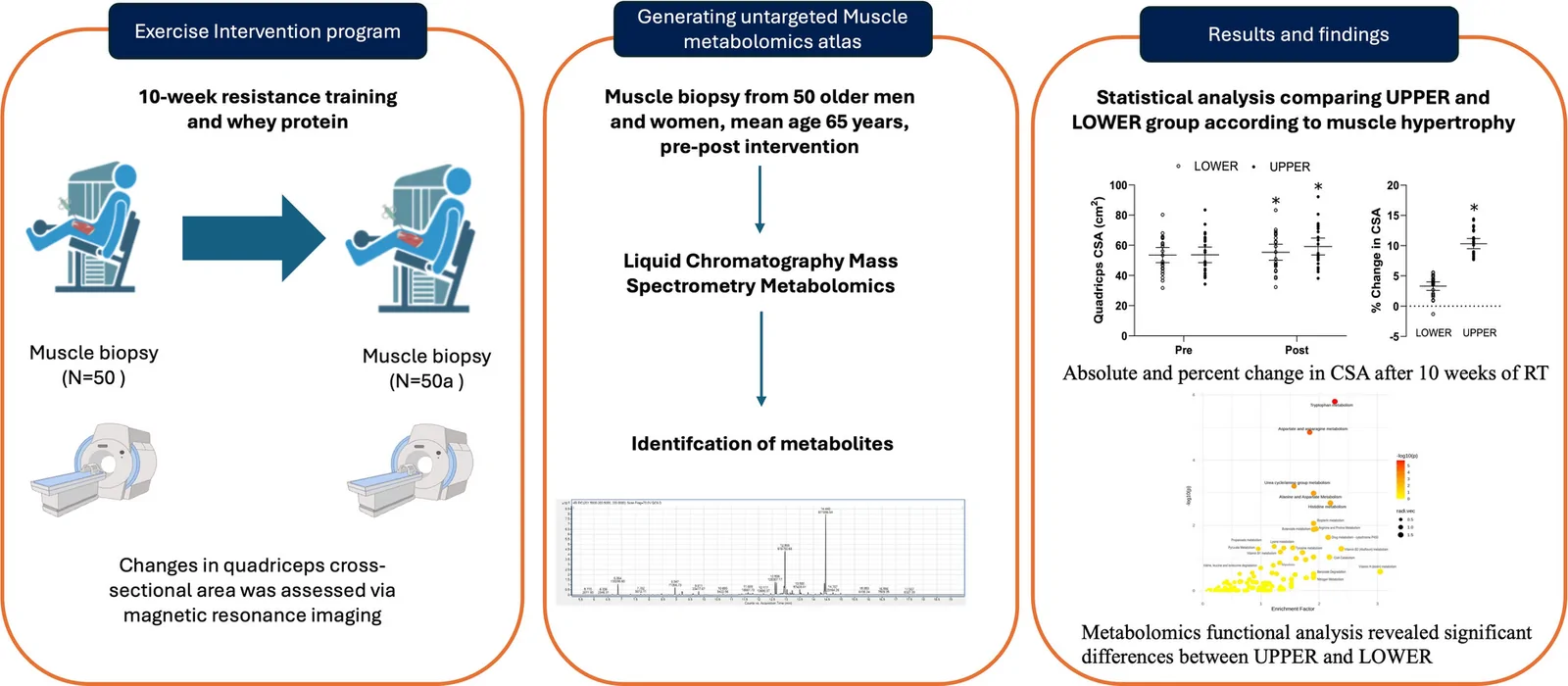

**Supplementary Information:**

The online version contains supplementary material available at https://doi.org/10.1007/s11357-025-02074-x.

## Introduction

The age-related loss of skeletal muscle mass is closely linked to frailty, loss of independence, and increased risk of comorbidities [1]. Thus, maintaining or increasing muscle mass is critical to support healthy ageing and extend healthspan. Among non-pharmacological strategies, resistance training (RT) combined with adequate protein intake is the most effective intervention for promoting muscle hypertrophy [2]. In particular, RT provides a potent anabolic stimulus by activating a range of endogenous skeletal muscle processes that lead to hypertrophic adaptations [3].

Although RT is effective in mitigating muscle loss, it is increasingly recognised that interindividual differences in endogenous responses play a critical role in determining the magnitude of hypertrophy [3]. Indeed, hypertrophic responses to RT vary widely among individuals, including those observed in older persons [4, 5]. Previous studies have reported that individuals exhibiting greater hypertrophy show higher androgen receptor content [6], greater satellite cell proliferation [7], and ribosome biogenesis following RT [8]. Moreover, transcriptomic analyses indicate that lower hypertrophy responders display differential transcriptomic responses in muscle following RT [9], reflecting altered molecular responsiveness, as well as substantial baseline differences in transcriptomic profiles among those with differing hypertrophic responses [10]. These findings highlight the importance of understanding the molecular and metabolic determinants underlying this variability in RT adaptations.

The skeletal muscle metabolome is an individualised reflection of both endogenous factors, including the transcriptome and proteome, and their interaction with exogenous influences, such as RT [11, 12]. Circulating metabolites have also been identified as strong predictors of ageing and disease [13, 14]. Thus, metabolomics may provide an approach to capture metabolic changes that may explain variability in hypertrophic responsiveness. Untargeted metabolomics, in particular, enables comprehensive profiling of small molecule metabolites, offering insights into pathways that regulate muscle adaptation. While prior studies have reported broad metabolic shifts with RT [12, 15], the effects of longer-term RT combined with protein supplementation on skeletal muscle metabolome in older adults remain poorly understood. Addressing this gap is critical, as it may provide insight into the underlying heterogeneity of response to RT, which could be used to gain insight into improve muscle health.

Therefore, we investigated skeletal muscle metabolic profiles before and after 10 weeks of RT in older adults, with participants classified as the upper (UPPER) and lower tertiles (LOWER) of RT-induced muscle hypertrophy. We hypothesised that UPPER and LOWER would exhibit distinct metabolic adaptations to RT.

## Methods

### Participants

We included 67 healthy older adults from our parental trial [16], comprising 30 males (68 ± 4 years; BMI = 26.4 ± 3.0 kg/m^2^) and 37 females (68 ± 5 years; BMI = 26.4 ± 4.3 kg/m^2^). Participants were generally healthy but had not engaged in regular exercise (resistance or aerobic training) for at least six months prior to the study. Exclusion criteria included type I diabetes, ischemic myocardial disease, arrhythmia, uncontrolled hypertension, and any major orthopaedic issues or musculoskeletal disorders. All participants provided written informed consent prior to enrolment and agreed to have their tissue samples stored and analysed after completion of the main study for secondary analysis.

### Ethics statement

The study was approved by the Human Research Ethics Committee of the University of Sao Paulo. All participants provided written, informed sent prior to taking part in the study, and agreed to have their tissue samples stored and analyzed after the main study. The main trial was registered at ClinicalTrials.gov (NCT number: NCT06718712). The ethical approval for muscle biopsy analysis was obtained from the University of Liverpool Central University Research Ethics Committee, reference number 12689.

### Experimental design

Participants completed 10 weeks of unilateral RT, with three supervised leg extension sessions per week (see *Resistance exercise training*). Quadriceps cross-sectional area (CSA) was measured by magnetic resonance imaging (MRI) pre- and post-training to assess hypertrophy. Muscle biopsies from the vastus lateralis were obtained pre- and post-RT, following MRI scans, and used for untargeted metabolomics analyses to compare UPPER vs. LOWER. During the RT intervention, participants consumed 20 g of whey protein twice daily (after breakfast and before sleep) to support muscle growth (> 1.2 g/kg/day) [17]. Participants were classified based on the technical error (TE) of MRI-derived changes in quadriceps CSA (see *Classification of responders)*.

### 1 repetition maximum test

Unilateral 1RM for knee extension was assessed according to established guidelines [9]. Briefly, following a 5 min cycle warm-up, participants completed 8 repetitions at 50% estimated 1RM and 3 repetitions at 70%, each separated by 1 min rest. After a 3 min recovery, up to 5 attempts were permitted to achieve 1RM, with 3 min rests. A repeated 1RM test was conducted 72 h later; the highest load was recorded as the final 1 RM.

### Resistance exercise training

As described in the parental study, participants trained one leg with 1 set and the contralateral leg with 4 sets of knee extensions (8–15 repetitions per set; 60–90 s of rest between sets). The trained leg was randomly assigned to minimise bias. For this study, only the multiset condition (4 sets) was analysed, as it is most strongly associated with hypertrophy [18]. All exercise sets were performed to volitional failure, and loads were adjusted to maintain the 8–15RM range. A certified exercise physiologist supervised all sessions.

### Quadriceps cross-sectional area

Quadriceps CSA was assessed using MRI (Sigma LX 9.1, GE Healthcare, Milwaukee, WI). Participants were positioned supine in the MRI scanner with their knees fully extended and secured using Velcro straps to ensure consistent positioning. Images were acquired at 50% of thigh length, defined as the midpoint between the greater trochanter and lateral epicondyle of the femur. Muscle CSA was analysed using ImageJ software (version 1.53c, National Institutes of Health, Bethesda, MD) by a blinded researcher. The reproducibility of MRI-derived CSA was assessed in 20 participants who underwent two scans, 7 days apart, prior to training [16]. The TE of the measurement was calculated as the standard deviation (SD) of repeated measures [19] and expressed as a coefficient of validation (1.63%).

### Classification of response

Hypertrophic responses were defined using the TE from repeated CSA scans. A change of greater than 2% in CSA was required to exceed measurement error. Higher responses (i.e., UPPER) were defined as persons showing > 7.7% CSA increase (greater than 3-times the TE), and lower responses (i.e., LOWER) as < 5.6%. (less than 2-times the TE). This classification led to the creation of two response groups, one with a higher response and the other with a lower response, each encompassing 25 participants, or ~ 37% (or one tertile) of the total participant sample from which muscle samples were taken, and for which muscle tissue was still available for analysis. The participants in the intermediate response range (5.6–7.7%) were classified as mid-range and were not analysed. Due to tissue and resource constraints, many of these participants were also excluded. This approach may have, we acknowledge, led to some bias; however, as a pragmatic first step in identifying metabolomic profiles associated with response heterogeneity, we viewed our method of comparing two distinct groups with a threefold difference in hypertrophic responses to RT as a reasonable preliminary investigative step, especially given our tissue and resource constraints.

### Muscle biopsy

Vastus lateralis biopsies were collected after an overnight fast (> 10 h) pre- and post-RT (48 h after the final training session). Participants were instructed to avoid vigorous physical activity for at least 72 h and to refrain from consuming alcohol and caffeine for 24 h prior to sampling. Biopsies were performed using a 5-mm Bergström needle adapted for manual sectioning under local anaesthesia (1% Xylocaine). A small incision was made, and ~ 150 mg of muscle tissue was collected. The muscle tissue was divided into aliquots (~ 25 mg), immediately (less than 60 s) frozen in liquid nitrogen, and stored at −80 °C until analysis.

### Untargeted liquid chromatography-mass spectrometry (LC–MS) metabolomics analysis

Muscle samples (~ 10 mg ± 1 mg) were suspended in 216 µL of pre-chilled MeOH:H_2_O (2.86:1) and homogenised in 2 mL Precellys™ tubes using a Qiagen PowerLyzer® 24 (5000 rpm, 2 × 10 s). Homogenates were transferred to 2 mL Eppendorf tubes, mixed with 240 µL of pre-chilled CHCl_3_:H_2_O (2:1), and incubated on ice for 10 min. Samples were vortexed for 30 s and centrifuged (3500 g, 4 °C, 5 min) to achieve phase separation. Aliquots (200 µL) of the polar (upper) and non-polar (lower) phases were collected into separate tubes and dried by evaporation in a cold-trap vacuum centrifuge. The dried extracts were stored at—80 °C until UHPLC-MS analysis. Optima® LC–MS grade MeOH, CHCl_3_, ACN, IPA and H_2_O were used for sample preparation, with LiChropur™ ammonium formate and formic acid (≥ 99.0%) as solvent buffers, and HPLC-grade absolute ethanol (99.8% v/v) was used for maintenance and cleaning.

### Quality control and assurance (QA/QC)

A fixed volume of homogenised tissue extract from each sample was pooled to generate both quality control (QC) and conditioning QC samples, following established protocols [20]. Pooled QC samples were analysed to monitor data quality, while extraction blanks, solvent blanks, QC samples, and system suitability test (SST) samples were prepared in parallel with study samples using the same preparation procedures [20].

### UHPLC-MS analysis

Untargeted metabolomics data were acquired using a ThermoFisher Scientific Vanquish UHPLC system coupled to an Orbitrap ID-X Tribridge MS (ThermoFisher Scientific Inc., UK). Dried polar extracts were reconstituted in 100 µL ACN:H2O (90:10), and the non-polar extracts in 100 µL H_2_O:MeOH (80:20), before transfer to glass vials. Polar extracts were separated on a Hypersil GOLD™ aQ C18 column (2.1 × 100 mm × 1.9 µm), while non-polar extracts were separated on a Hypersil GOLD™ Vanquish C18 column. During analysis, columns were maintained at 50 °C with a 15 min gradient elution at a flow rate of 0.4 mL/min. Samples were stored at 4 °C in the LC autosampler, and 5 µL was injected for both positive and negative electrospray ionisation (ESI). Data were acquired in full-scan mode. Each analytical batch was bracketed with blank and conditioning QC injections and included periodic pooled QC sample injections [21]. For compound annotation and identification, ddMS2 data were acquired from 3 pooled QC samples across four m/z ranges: (a) 66.7–1000, (b) 66.7–300, (c) 300–600, and (d) 600–900 [22]. Detailed gradient elution profiles, solvent compositions, and MS parameters are provided in Supplemental Tables 1 and 2, respectively.

**Table 1:** Baseline characteristics

	**LOWER (*n* = 25)**	**UPPER (*n* = 25)**	***P***
Sex (M/F)	10/15	13/12	
Age (yr)	69 ± 5	67 ± 4	0.086
Body mass (kg)	71 ± 13	72 ± 13	0.745
Height (m)	1.62 ± 0.1	1.66 ± 0.1	0.363
Body mass index (kg/m^2^)	27 ± 4	26 ± 3	0.829
1 repetition maximum (kg)	42 ± 16	51 ± 21	0.224
Clinical conditions			
Type 2 diabetes, *n* (%)	1 (4)	2 (8)	0.837
Hypertension, *n* (%)	7 (28)	4 (16)	0.579
Cholesterol, *n* (%)	7 (28)	5 (20)	0.264

Data are expressed as mean (SD)

*LOWER* lower tertile response, *UPPER* upper tertile response; *T2DM *type 2 diabetes

### Quantification and statistical analysis

Normality was tested using the Shapiro–Wilk test. Baseline characteristics between the UPPER and LOWER groups were compared using an unpaired t-test, and nominal variables (i.e., clinical conditions) were compared using the Chi-square test. Absolute quadriceps CSA changes were analysed by linear mixed models (group x time), with Tukey post hoc test. Percent CSA changes were compared using an unpaired t-test. Statistical significance was set at P < 0.05, and results are reported as mean ± SD. Analyses were performed in R (version 4.3.2). The LC–MS data were deconvolved using Compound Discoverer 3.2 (Thermo-Fisher), with tentative annotation based on MS/MS spectral matching. Deconvolved data were imported into MATLAB (2023a, Mathworks, MA) for multivariate analysis. Partial least squares–discriminant analysis (PLS-DA) was used to assess separation (UPPER vs. LOWER; pre- vs. post-RT). The number of PLS components was determined by using a sevenfold cross-validation on the training set. The optimal number of PLS components was set to the ones that had the best classification accuracies on the validation sets. The exact optimal number of components varied, depending on the combination of training and test set, with most PLS models selecting 2–3 PLS components as optimal. Variable Importance in Projection (VIP) scores identified key discriminating metabolites (VIP > 1). Pathway enrichment was performed using the Mummichog algorithm and the Kyoto Encyclopaedia of Genes and Genomes (KEGG) database in MetaboAnalyst (version 6.0). Full metabolite lists are provided in Supplemental Data 1. These data also included results from N-way ANOVA, and p-values for the baseline and pre-post comparisons in the UPPER and LOWER groups, and were not adjusted for covariates. Model performance was validated through 1,000 bootstrapping iterations (23), and statistical significance was further confirmed via permutation testing. Metabolite lists were manually curated to distinguish biological relevant signals from noise. Finally, N-way ANOVA was applied to log-transformed features to detect group (UPPER vs. LOWER) and time (pre- vs. post-RT) effects, with Benjamin-Hochberg false discovery rate (FDR) correction for multiple testing.

## Results

### Participant characteristics

Baseline characteristics are presented in Table 1. No significant differences were observed between LOWER and UPPER in anthropometric measures, clinical conditions (type 2 diabetes, hypertension, and hypercholesterolemia) prior to the intervention.

### Quadriceps muscle cross-sectional area

**Fig. 1:**
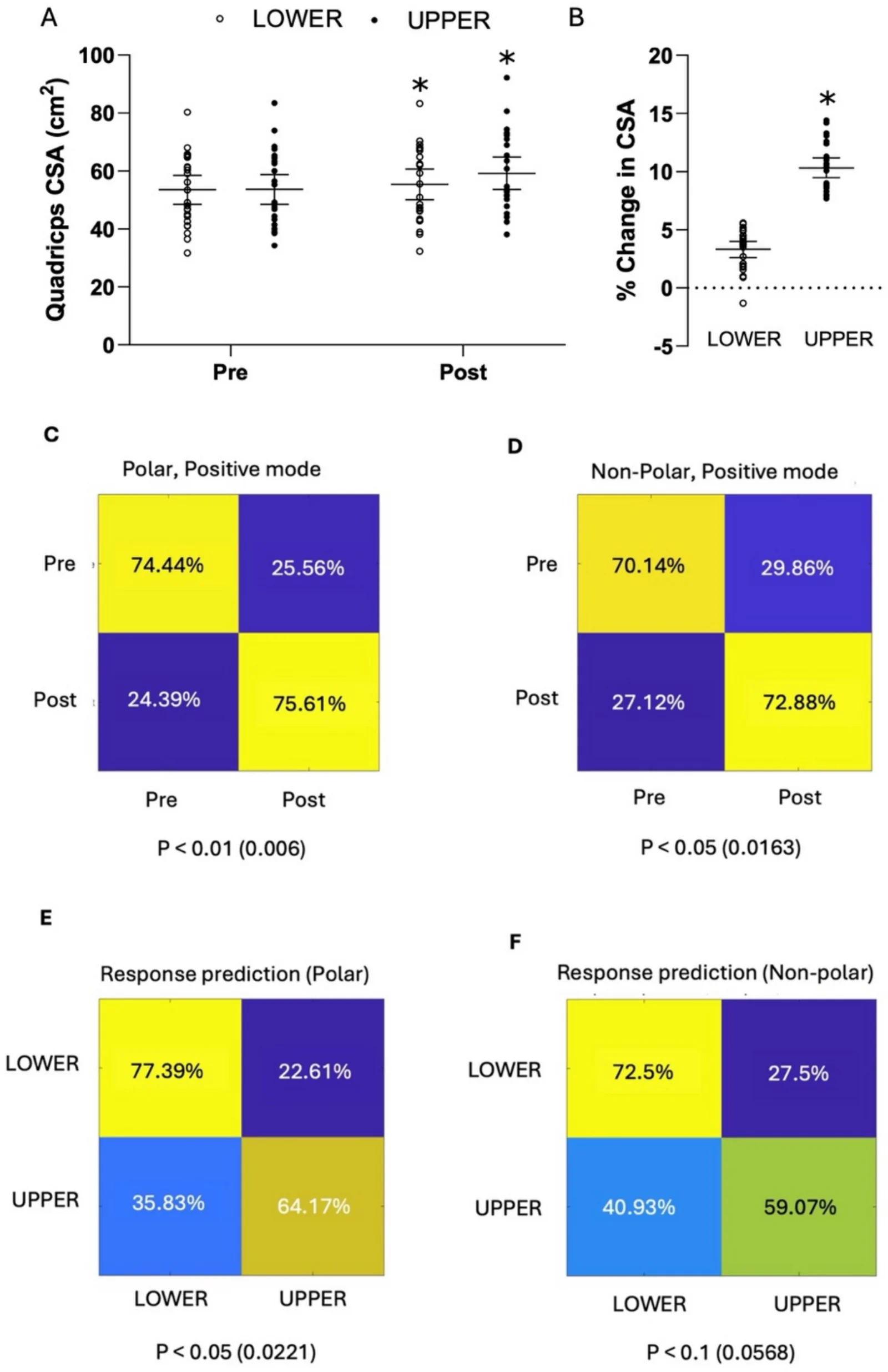
**A** Absolute quadriceps muscle CSA pre- and post-intervention (each dot is an individual value; shown with mean and 95% confidence interval), **B** and percent change in CSA after 10 weeks of RT (each dot is an individual value; shown with mean and 95% confidence interval), Partial Least-Squares Discriminant Analysis (PLS-DA) results are shown for **C** polar positive mode and **D** non-polar positive mode in pre- and post-intervention from all participants, and for **E** polar positive mode and **F** non-polar positive mode comparing UPPER and LOWER groups. LOWER, lower tertile response; UPPER, upper tertile response. * P < 0.05, significant difference from pre-intervention within groups; (**A**), and * P < 0.001, significant difference between groups (**B**)

In the LOWER group, absolute quadriceps CSA increased from 53.6 ± 12.1 cm^2^ to 55.4 ± 12.8 cm^2^ after 10 weeks of RT (3.3 ± 1.7%, P < 0.001; Fig. 1A). In the UPPER group, CSA increased from 53.7 ± 12.5 cm^2^ to 59.2 ± 13.6 cm^2^ (10.3 ± 2.0%, P < 0.001; Fig. 1A). Regarding changes in CSA between pre and post, LOWER and UPPER increased by 3.3% and 10.3%, respectively (Fig. 1B). UPPER showed a significantly greater percentage change in CSA compared with the LOWER group (P < 0.001; Fig. 1B).

### Metabolic profile of skeletal muscle

Untargeted LC–MS analysis detected > 2,500 metabolites across skeletal muscle samples, which were mapped to 104 metabolic pathways. PLS-DA using polar extracts provided the highest discrimination between UPPER and LOWER, with an average correct classification rate (CCR) of 75% (Fig. 1C). The empirical P-value was 0.006, indicating that only 6 out of 1,000 permutation tests produced null models with better performance. Non-polar extracts achieved a slightly lower CCR at 72% (P = 0.0163; Fig. 1D). In general, classification performance was higher for pre- vs. post-RT than for UPPER vs. LOWER (Fig. 1E and F).

### Mummichog functional enrichment analysis

**Fig. 2:**
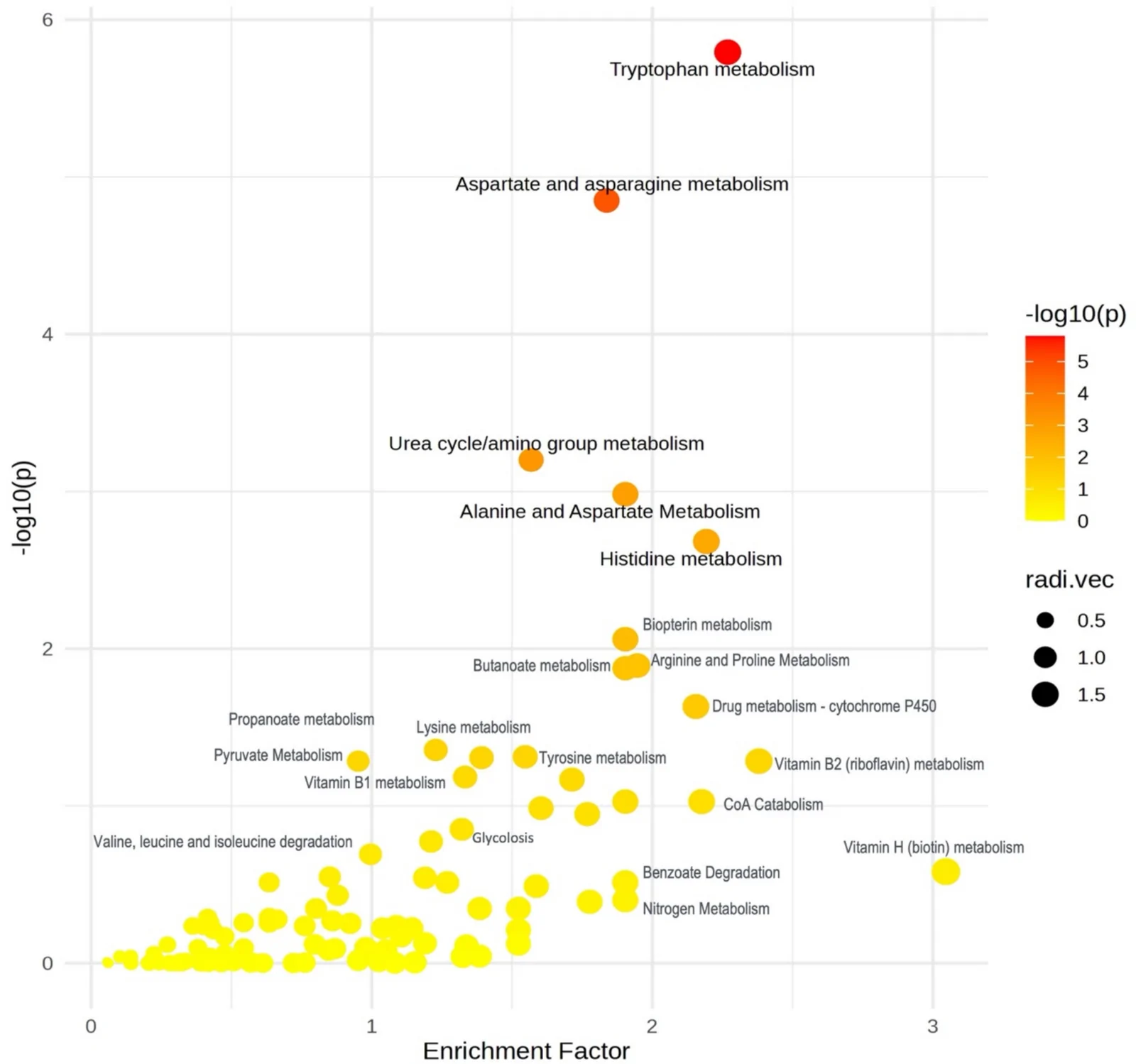
Results from the Mummichog functional analysis based on UPPER compared with LOWER groups to the intervention. Unique identified m/z values, t scores, and p values derived from two-way ANOVA were utilised to generate the enriched pathways. A total of 105 metabolic pathways were mapped based on the MFN human genome-scale metabolic model. The most enriched pathways are displayed, with nodes distributed across the enrichment factor and a heatmap illustrating the significance level

For the functional analysis, we focused on the comparison between UPPER and LOWER responses to intervention as the most significant model (Fig. 2) (Supplemental Data 2). Pathway enrichment analysis using Mummichog identified several significantly altered metabolic pathways following RT. The most significantly enriched pathway was tryptophan metabolism (P (Fisher) < 0.0001), followed by aspartate and asparagine metabolism (P (Fisher) < 0.0001) and urea cycle/amino group metabolism (P (Fisher) < 0.0001). Additional pathways showing significant enrichment included alanine and aspartate metabolism (P (Fisher) = 0.0010, FDR = 0.0448), histidine metabolism (P (Fisher) = 0.0021), biopterin metabolism (P (Fisher) = 0.0087), arginine and proline metabolism (P (Fisher) = 0.0128), and butanoate metabolism (P (Fisher) = 0.0133). Other enriched pathways included drug metabolism–cytochrome P450 (P (Fisher) = 0.0233), propanoate metabolism (P (Fisher) = 0.0441), and tyrosine metabolism (P(Fisher) = 0.0488).

### Differential metabolites between UPPER and LOWER

**Fig. 3:**
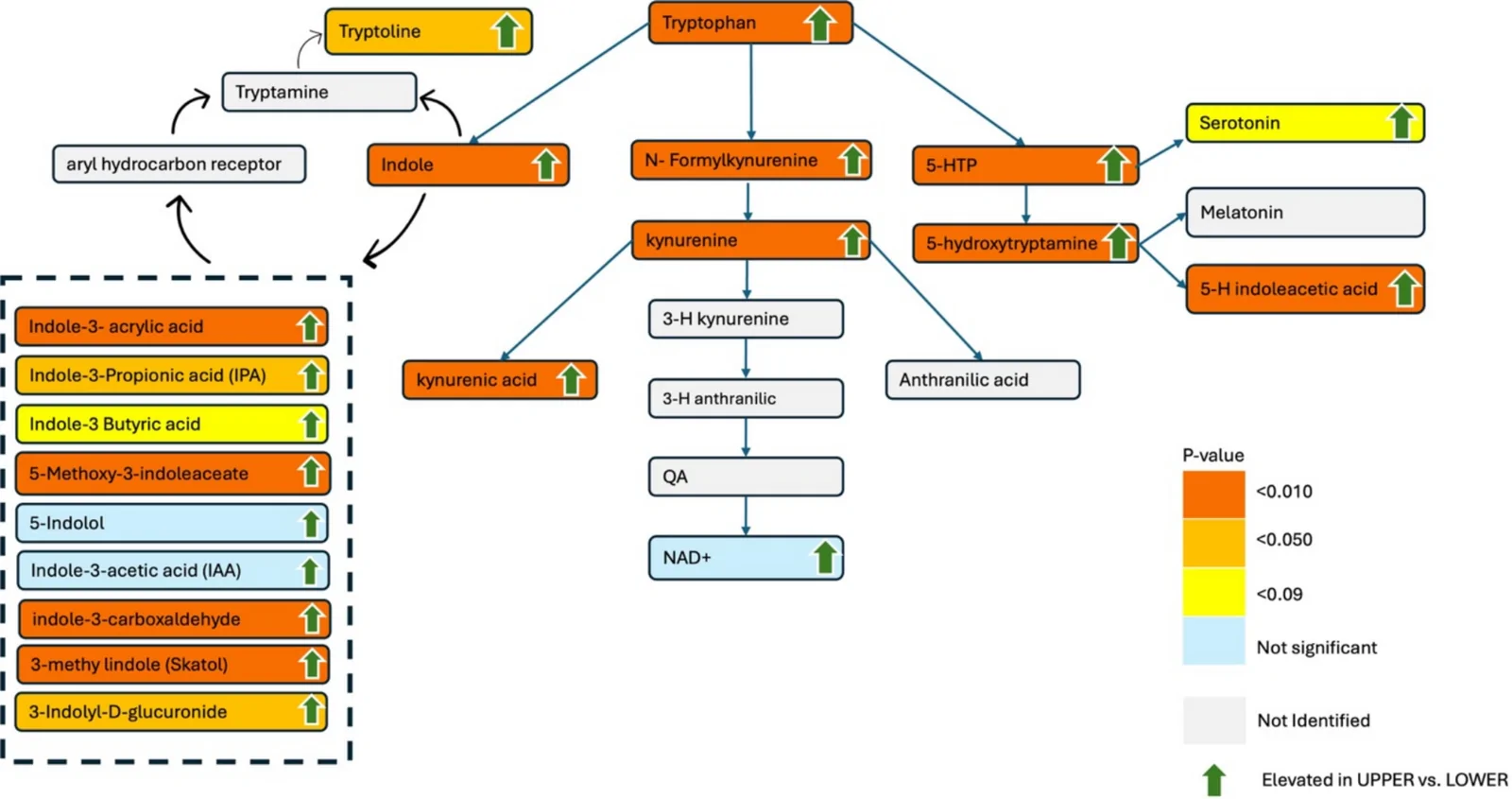
Schematic overview of tryptophan metabolism pathways and the metabolites detected in UPPER group vs LOWER group

**Fig. 4:**
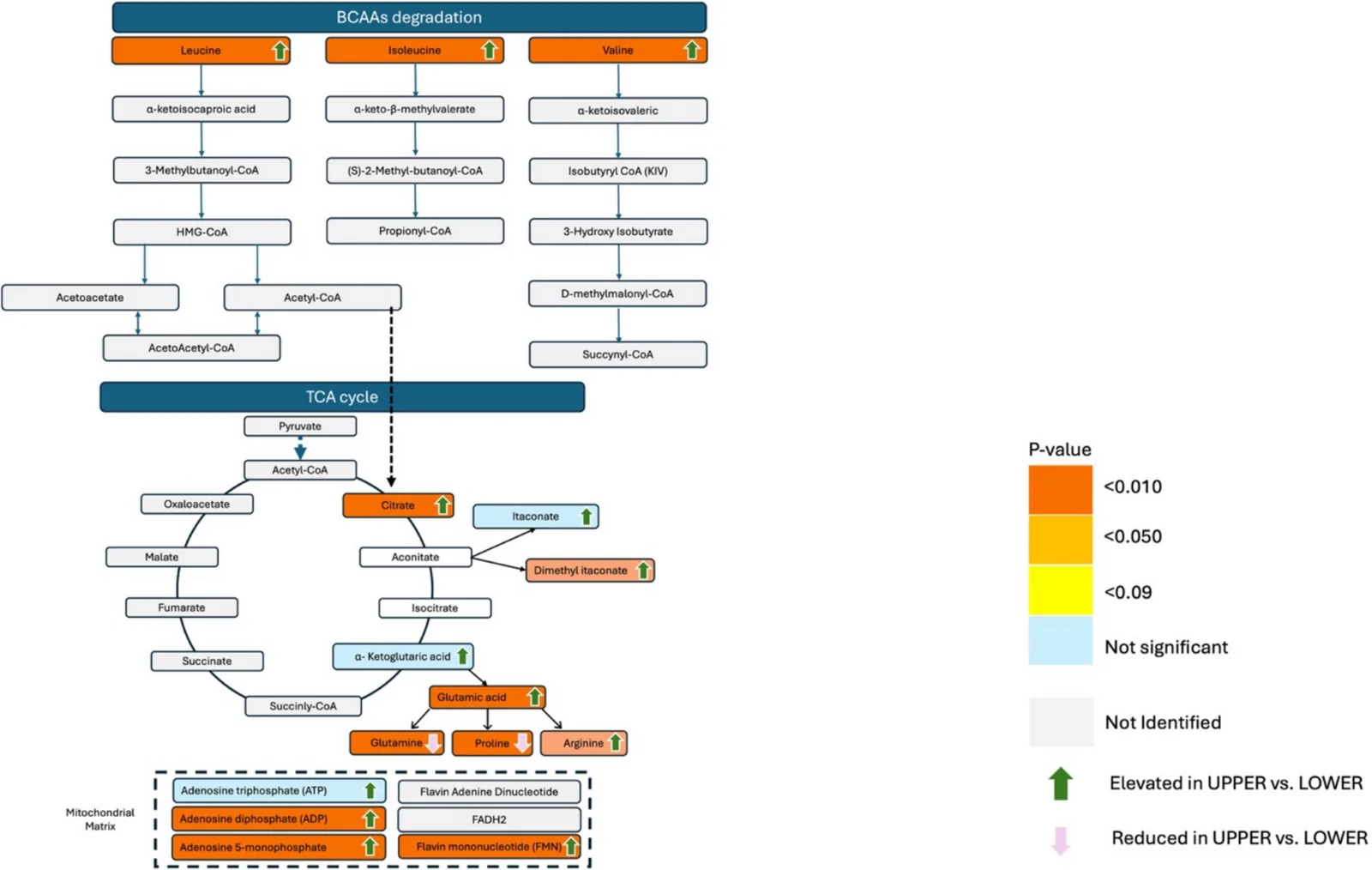
Schematic overview of BCAA degradation interconnection with the Tricarboxylic acid (TCA) intermediate pathways and the metabolites elevated or reduced in UPPER vs. LOWER

We have provided results for all four possible comparisons in Supplemental Data 2, which includes pre- and post-RT comparisons between the UPPER and LOWER groups. Here we present the results and interpretation for the most statistically significant model, in line with the functional analysis. Compared with the LOWER, the UPPER group showed broad upregulation of amino acid- and peptide-related metabolites (Supplemental Data 2). Tryptophan-related metabolites were upregulated in the UPPER, including tryptophan, indole derivatives, kynurenine, kynurenic acid, as well as 5HT (Fig. 3). In addition, several metabolites from vitamin and cofactor metabolism (e.g., riboflavin and nicotinamide), and histidine metabolism were upregulated in the UPPER compared with the LOWER group (Supplemental Data 2). From the BCAA pathway, valine, leucine and isoleucine were upregulated in the UPPER compared with the LOWER, whereas no BCAA catabolites were identified (Fig. 4A). Among the TCA intermediates, citrate was upregulated in UPPER compared with LOWER, while other intermediates, including succinate, fumarate, malate, oxaloacetate, succinyl-CoA, and aconitate, were not detected. Itaconate and ATP were detected but showed no significant changes. Furthermore, amino acids linked to the TCA cycle (glutamic acid, glutamine, proline, and arginine) were elevated in UPPER. Additionally, energy-related metabolites, including adenosine diphosphate, adenosine monophosphate, were increased (Fig. 4B), contributing to enriched urea and amino acid group metabolism (Supplemental Data 1).

## Discussion

We identified and mapped metabolic pathways and potential metabolic signatures of RT-induced skeletal muscle hypertrophy in older adults. We observed distinct muscle metabolic adaptations between UPPER and LOWER after 10 weeks of RT, with UPPER characterised by enhanced amino acids, especially tryptophan pathways, implicating the potential metabolic signatures associated with hypertrophic responsiveness in older adults. Importantly, the participants showed no baseline differences in phenotype or metabotype that distinguished the groups.

Our metabolomic profiling data extend the main study findings [16] by providing biochemical context to the variability in hypertrophic responsiveness among older adults. In the original study, higher training volume effectively mitigated nonresponse, demonstrating that some older adults require a greater stimulus to activate anabolic pathways. For that reason, we chose to classify response in the leg that completed 4 sets and not 1. The companion transcriptomic study [9], revealed that robust hypertrophy in high responders was accompanied by upregulation of genes involved in amino acid metabolism, protein turnover, and protein folding, whereas low responders exhibited minimal molecular plasticity. The current metabolomic analysis complements these findings [9] by showing that individuals with greater hypertrophy displayed elevated intramuscular levels of tryptophan–kynurenine metabolites, branched-chain amino acids, and urea-cycle intermediates, consistent with efficient amino acid utilization and mitochondrial energy regulation. These overlapping molecular signatures suggest that enhanced amino acid metabolism and improved mitochondrial energetics may be key features associated with hypertrophic responsiveness. Together, the converging metabolomic and transcriptomic data reinforce the concept that higher RT volume promotes metabolic and molecular adaptations that favour anabolic efficiency in older adults[9], offering mechanistic insight into how RT variables can offset age-related heterogeneity in muscle growth. This interpretation is broadly consistent with large scale scoping analyses of RT variables that affect hypertrophy[18]. Collectively, our and the previous studies also show that with aging there is still a retention of the capacity[9, 18], with a sufficient RT stimulus, to achieve robust hypertrophy that is not characterized by non-response, but still a pronounced variability in response to RT.

Functional analysis and pathway mapping highlighted significant enrichment of tryptophan metabolism in UPPER compared with LOWER (Fig. 3). Tryptophan is an essential amino acid that is degraded by enzymes into kynurenines [23]. Consistent with our findings, previous work reported higher muscle kynurenine levels in active than with sedentary older adults [24]. Importantly, downstream metabolites such as kynurenic acid and nicotinamide adenine dinucleotide (NAD^+^) have been associated with improved respiratory fitness and enhanced muscle oxidative capacity [25]. Moreover, tryptophan catabolism is influenced by the gut microbiome, particularly via indole-derived metabolites [26–28]. Indole metabolites, such as indole-3-propionic acid and indole-3-acetic acid, have been shown to protect against inflammation and promote nerve regeneration in vitro [28, 29], and to contribute to muscle growth and metabolic function in vivo [30–33]. The abundance of gut-derived indoles may imply an association of the gut-muscle axis with hypertrophic regulation in older adults. However, the mechanism of action of indoles in human muscle remains unclear, and further mechanistic studies are warranted.

Enrichment of aspartate and asparagine metabolism, along with the urea cycle, reflects robust amino acid and TCA cycle activity, given that key metabolites from these pathways include glutamic acid, α-ketoglutaric acid, proline, aspartic acid, spermidine, carnitine, arginine, carnosine, acetylcarnitine, and lysine. A comprehensive interpretation of all metabolites within these pathways is beyond the scope of this study; therefore, we focus on those that are most biologically relevant and central to metabolic adaptations to exercise. Carnosine was elevated in UPPER compared with LOWER (Supplemental Data 1). Carnosine is synthesised from β-alanine and histidine and found in high concentrations in skeletal muscle. Alongside carnitine, it contributes to pH buffering, ATP regeneration, and energy availability [34, 35], all of which are essential for adaptation to RT and may partly explain the greater hypertrophic capacity of UPPER. Supporting this thesis, Hoetker et al. [36] reported dynamic changes in mitochondrial carnitine homeostasis and ATPDG1 expression during exercise, implying a role for carnosine in regulating carnitine balance. Our findings suggest enhanced carnosine synthesis may stabilise carnitine levels in UPPER, potentially improving energy efficiency, which is essential for muscle protein synthesis in UPPER.

RT also altered muscle acylcarnitine levels, with higher acetylcarnitine (C2) and other short- and medium-chain acylcarnitines in UPPER. These metabolites are central to transporting acyl groups (organic acids and fatty acids) from the cytoplasm into mitochondria, where they undergo oxidation to produce energy [24, 37]. Our findings are consistent with previous reports, showing that exercise induces the upregulation of acylcarnitines, thereby modulating muscle bioenergetics and acetyl group balance during and after exercise [38]. Key acylcarnitines identified in this study include C2, a short-chain metabolite central to energy metabolism, and 3-hydroxyoctanoylcarnitine, a medium-chain hydroxylated acylcarnitine indicative of incomplete fatty acid β-oxidation (Supplemental Data 1). Additionally, medium-chain acylcarnitines such as O-heptanoyl carnitine and octanoyl-carnitine play crucial roles in the carnitine shuttle [37].

Further, the metabolic mapping of BCAAs (Fig. 4A) showed an upregulation of leucine, valine, and isoleucine in the UPPER. However, BCAA catabolites were not detected. This absence may reflect an overall improvement in BCAA metabolism, preventing the accumulation of downstream catabolites. Notably, as the majority of BCAA-derived carbons ultimately enter the TCA cycle, our finding showed an increase in citrate, α- Ketoglutaric acid, glutamic acid, glutamine, proline, and arginine. These interconnected findings, although observational, may suggest that BCAA- and TCA-related intermediates were not accumulating excessively. This interpretation is further supported by increases in adenosine diphosphate and adenosine 5-monophosphate, indicating robust energy production [39]. Nevertheless, validation of this interpretation would require targeted metabolomic analysis, and serial muscle biopsies to enable metabolic flux assessment. In addition, cross-sectional analyses integrating metabolomics and transcriptomics have suggested impaired BCAA catabolism as a potential mechanism underlying ageing-related muscle loss [40]. Overall, the downstream BCAA degradation in response to RT remains an understudied pathway and warrants further investigation.

Muscle biopsies were collected 48 h post-training, reflecting medium- to longer-term adaptations rather than an acute response [41]. Limited tissue availability prevented confirmatory targeted analyses. Sex-specific effects could not be examined due to sample size, although both sexes were represented in the UPPER and LOWER groups. Future work incorporating analyses of circulating metabolomes and faecal microbiota could provide a more comprehensive view of RT adaptations. Hypertrophic responses were defined using the TE from repeated MRI scans to determine muscle CSA. Both the UPPER and LOWER groups showed significant (versus baseline) hypertrophy, so there was no true ‘non-response’ to RT. By analysing only these groups, we acknowledge that this approach may have introduced bias; however, as an ancillary analysis of the original study data, it represented a reasonable first step toward identifying metabolic pathways associated with variability in hypertrophic responses. Regrettably, tissue availability and resource constraints prevented analysis of the complete participant sample and may limit robust comparison with the parent data. Clearly, larger studies encompassing the full spectrum of metabolomic responses and relating changes across the entire range will be required to validate and extend our findings.

We identified distinct skeletal muscle metabolomic signatures between UPPER and LOWER in older adults, highlighting enhanced metabolic pathways related to essential amino acids, mitochondrial metabolism, and the gut in UPPER. These findings offer insight into the molecular determinants that may underpin interindividual variability in RT-induced hypertrophy in older adults, informing strategies to optimise training outcomes and mitigate age-related muscle decline.

## Supplementary Information

Below is the link to the electronic supplementary material.

Supplementary file1 (DOCX 29 kb)

Supplementary file2 (CSV 6 kb)

Supplementary file3 (XLSX 1817 kb)

## Data Availability

Most of the data supporting the findings of this study are available in the supplemental materials.
