# Supplementary material for: Skeletal muscle metabolomic markers underlying the enhanced exercise-induced hypertrophy response to resistance training in older adults
Source: GeroScience. 2026 Jan 5;48(6):7693–705. doi: 10.1007/s11357-025-02074-x (PMC13638854; doi:10.1007/s11357-025-02074-x)
Supplement: Supplementary file 1 [file 11357_2025_2074_MOESM1_ESM.docx]

# Supplemental Table 1. Ultra-high performance Liquid Chromatography conditions.

| **UHPLC** | **Polar** | | **Non-polar** | |
| --- | --- | --- | --- | --- |
| Flow rate | 0.4 mL/min |  |  |  |
| Buffer composition | 0.1% Formic Acid | | 10mM Ammonium Formate  and 0.1% Formic Acid | |
| Solvent composition | % (A) | % (B) | % (A) | % (B) |
| Time (min) | Gradient curve = 5, linear | | | |
| 0 | 80 | 20 | 80 | 20 |
| 1.60 | 80 | 20 | 80 | 20 |
| 9.40 | 100 | 0 | 100 | 0 |
| 10.60 | 100 | 0 | 100 | 0 |
| 12.60 | 80 | 20 | 80 | 20 |
| 15.00 | 80 | 20 | 80 | 20 |
| 15.00 | Gradient end | | | |

UHPLC, Ultra-high performance Liquid Chromatography.

# Supplemental Table 2. Orbitrap ID-X Tribid mass spectrometry parameters.

| **MS parameter** | **Polar (ESI+)** | **Polar (ESI-)** | **Nonpolar (ESI+)** | **Nonpolar (ESI-)** |
| --- | --- | --- | --- | --- |
| m/z range | 66.7 – 1000 | 66.7 – 1000 | 150 – 2000 | 150 – 2000 |
| Resolution  (FWHM at m/z 200) | 120,000 | | 60,000 | |
| Spray voltage (kV) | 3.2 | 3.0 | 3.2 | 2.7 |
| Sheath Gas (AU) | 40 | | 40 | |
| Aux Gas (AU) | 8 | | 8 | |
| Sweep Gas (AU) | 1 | | 1 | |
| Capillary Temp (°C ) | 275 | | 300 | |
| ESI Heater Temp (°C ) | 320 | | 350 | |
| Max. Injection time (s) | 100 | | 50 | |

MS, mass spectrometry; ESI, electrospray ionisation; FWHM, full width at half maximum.
